# Gender differences in the association of dementia symptoms severity and hospital anxiety

**DOI:** 10.1192/j.eurpsy.2024.467

**Published:** 2024-08-27

**Authors:** K. Bosak, S. Vuk Pisk, M. Grah, P. Folnegović Grošić, Ž. Bajić, V. Grošić

**Affiliations:** ^1^Psychiatric Clinic Sveti Ivan; ^2^Department of Psychiatry, University Hospital Centre; ^3^Research Unit “Dr. Mirko Grmek”, Psychiatric Clinic Sveti Ivan, Zagreb, Croatia

## Abstract

**Introduction:**

Symptoms of anxiety can worsen cognitive decline in people with dementia, and symptoms of dementia and fears of further cognitive decline can cause anxiety. Advanced dementia is not necessarily associated with a loss of emotion, which means that people may still experience anxiety, but their ability to cope in a healthy way is reduced. The problem is exacerbated by the fact that hospital care alone can contribute to the severity of anxiety symptoms due to change in routine of daily activities, unfamiliar surroundings, uncertainty about health status, prognosis and various medical procedures. There are differences between women and men in the prevalence, course and manifestation of both dementia and anxiety disorders.

**Objectives:**

To examine whether there are differences between women and men in the association of hospital anxiety with eight symptoms of dementia: difficulties with memory, orientation, judgement, problem solving, fulfilling social obligations, daily activities at home and with hobbies, or personal care, in patients with mild to moderate dementia.

**Methods:**

A cross-sectional study was conducted at Sveti Ivan Psychiatric Clinic in Zagreb in June 2023. The target population were hospitalised patients diagnosed with dementia, both genderes, aged 60-90 years, without psychotic disorder. Anxiety during hospitalisation was measured using the anxiety subscale of the Hospital Anxiety and Depression Scale (HADS-A), and the severity of dementia symptoms was measured using the Dementia Assessment Instrument (CDR). The hypothesis was tested using Wald test of differences between women and men in unstandardised linear regression coefficients of the HADS-A on individual dementia symptoms, after adjustment for age, education, presence of a married or stable non-marital partner and duration of current hospitalisation.

**Results:**

We enrolled 65 women and 35 men of comparable age. There were significant gender-related differences in the association between hospital anxiety and difficulties with judgement (P = 0.01), fulfilling social obligations (P < 0.001), difficulties with home and hobbies (P = 0.02), and personal care (P = 0.00). In women, more pronounced difficulties with judgement and with home and hobbies were associated with higher anxiety, and in men the presence of these two symptoms of dementia was associated with lower anxiety. Difficulties with fulfilling social obligations are associated with lower anxiety in women and higher anxiety in men. Difficulties with personal care were associated with lower anxiety in both genders, but this effect was stronger in men.

**Conclusions:**

There are differences between women and men in the association between anxiety during hospital treatment and the severity of individual dementia symptoms. These differences are present in difficulties with judgement, fulfilling social obligations, home, hobbies and personal care.

**Disclosure of Interest:**

None Declared

